# Retinal Photoreceptors and Microvascular Changes in Prediabetes Measured with Adaptive Optics (rtx1™): A Case-Control Study

**DOI:** 10.1155/2017/4174292

**Published:** 2017-11-07

**Authors:** Anna Zaleska-Żmijewska, Paweł Piątkiewicz, Barbara Śmigielska, Anna Sokołowska-Oracz, Zbigniew M. Wawrzyniak, Dorota Romaniuk, Jerzy Szaflik, Jacek P. Szaflik

**Affiliations:** ^1^Department of Ophthalmology, SPKSO Ophthalmic Hospital, Medical University of Warsaw, Warsaw, Poland; ^2^Department of Internal Diseases, Diabetology and Endocrinology, Medical University of Warsaw, Warsaw, Poland; ^3^Retinopathy Outpatient Clinic, Masovian Hospital Bródno, Warsaw, Poland; ^4^Faculty of Electronics and Information Technology, Warsaw University of Technology, Warsaw, Poland; ^5^Department of Ophthalmology, SPKSO Ophthalmic Hospital, Warsaw, Poland

## Abstract

**Background:**

Patients with prediabetes are at risk for diabetes, cardiovascular events, and microvascular complications. The rtx1 (Imagine Eyes, France) permits early detection of changes in the retinal photoreceptors and vessels.

**Objective:**

Cone parameters and retinal microvasculature were analyzed with the rtx1 in 12 prediabetic patients and 22 healthy subjects. The analysis was based on cone density (DM), interphotoreceptor distance (SM), cone packing regularity, and retinal vessel parameters: wall thickness, lumen diameter (LD), wall-to-lumen ratio (WLR), and cross-sectional area of the vascular wall.

**Results:**

DM in the prediabetic group was not significantly lower than that in the control group (18,935 ± 1713 cells/mm^2^ and 19,900 ± 2375 cells/mm^2^, respectively; *p* = 0.0928). The LD and WLR means differed significantly between the prediabetic and the control groups (LD 94.3 ± 10.9 versus 101.2 ± 15, *p* = 0.022; WLR 0.29 ± 0.05 versus 0.22 ± 0.03, *p* < 0.05). A multivariate regression analysis showed that the WLR was significantly correlated with BMI and total cholesterol.

**Conclusions:**

Abnormalities found in rtx1 examinations indicated early signs of arteriolar dysfunction, prior to impaired glucose tolerance progressing to diabetes. The rtx1 retinal image analysis offers noninvasive measurement of early changes in the vasculature that routine clinical examination cannot detect.

## 1. Introduction

Diabetes is a chronic disease of great medical and socioeconomic consideration. Currently, there is an epidemic of diabetes, and the worldwide population of patients is expected to increase from 366 million in 2011 to 552 million in 2030 [1].

A complication associated with diabetes mellitus is diabetic retinopathy (DR), a chronic, progressive, and sight-threatening disease of the retinal microvasculature and neuronal cells [2, 3]. The development and progression of DR are closely associated with the type and duration of diabetes, glucose levels, and blood pressure.

Microvascular changes accompany or precede neuronal changes in the retina. In addition, evidence suggests that neural dysfunction occurs in diabetes before the appearance of microvascular lesions [4]. Neural degenerative changes in the retina that are associated with diabetes involve apoptosis of several populations of retinal cells, including retinal ganglion cells, which are especially sensitive to hypoxia, as well as photoreceptors and bipolar cells [3]. Microvascular abnormalities seen in early-to-moderate stages of DR include loss of pericytes, basement membrane thickening, arteriole wall thickening, and formation of microaneurysms [5]. During hyperglycemia, failure of Muller cell scavenging of glutamate from the extracellular space of the retina contributes to glutamate cytotoxicity of the retinal neurons [6]. The defect in the retinal glial cells in turn contributes to the early changes in the photoreceptors and retinal microvasculature because support for neuronal and vascular homeostasis deteriorates [6, 7]. Consequently, DR is a disease of both retinal neurons and microcirculation.

In 1997 and 2003, the Expert Committee on the Diagnosis and Classification of Diabetes Mellitus recognized prediabetes, a disorder in which blood glucose levels do not meet the criteria for diabetes but are higher than normal [8, 9]. Individuals with prediabetes have impaired fasting glucose (IFG), with fasting plasma glucose (FPG) ranging from 100 mg/dL (5.6 mmol/L) to 125 mg/dL (6.9 mmol/L), or impaired glucose tolerance (IGT), in which 2-h values in the oral glucose tolerance test (OGTT) range from 140 mg/dL (7.8 mmol/L) to 199 mg/dL (11.0 mmol/L) [8, 9]. Most people with prediabetes also have signs of metabolic syndromes, such as insulin resistance, dyslipidemia, obesity (especially abdominal or visceral), and hypertension. Individuals with IFG and/or IGT are at relatively high risk for the future development of diabetes and cardiovascular events [8, 9]. Prediabetes affects about 11% of people aged 50–59 and 14.2% aged 60–75 in the United States [10]. The number of individuals identified as having prediabetes is expected to rise to 400 million worldwide in 2030 [11].

Evidence suggests that IFG and IGT lead to both macrovascular and microvascular complications traditionally attributed to diabetes [12, 13]. Epidemiological studies indicate that vascular complications are more frequent in patients with insulin resistance, even before hyperglycemia develops [14]. Results of the AusDiab Study and the Diabetes Prevention Program showed that 6.7% to 7.9% of patients with IGT or IFG will develop retinopathy [15, 16].

The natural progression of DR to the late stages has been well characterized, but the early stages preceding a DR diagnosis have not been well established. Fluorescein angiography is the gold standard for visualizing human retinal vessels, but it is not typically performed in patients with diabetes who do not have typical signs of DR. Digital retinal photography and new imaging technologies now allow more precise visualization of subtle retinal changes. For example, the high reliability of retinal photography was proved in the Atherosclerosis Risk in Communities (ARIC) Study [17]. Hubbard and coworkers developed a semiautomated technique for the measurement of retinal vascular caliber from a series of photographs, described as the arteriovenous ratio [18].

Innovative optical technologies permit early detection of tissue changes. One noninvasive method for visualizing microcirculation and retinal structures is adaptive optics scanning laser ophthalmoscope. This application has been used in several studies on patients with diabetes, with a particular focus on capillary network topology [19–21].

The rtx1 (Imagine Eyes, Orsay, France) is the microscope which uses adaptive optics technology. It permits visualization of single retinal cells (photoreceptors) and the smallest blood vessels. The image resolution achieved by this technology is superior to that of any other current diagnostic tool. Adaptive optics allows capturing enface images of photoreceptors at near-histological resolution. The rtx1 camera uses an infrared illumination (wavelength of 850 nm) and has a resolution of 1.6 *μ*m. The field of view is 4° × 4°, which corresponds to an approximately 1.2 × 1.2 mm square on the retinal surface based on the axial length of the eye. The total image acquisition time is 4 s, during which 40 individual images can be acquired. The rtx1 microscope includes image acquisition and object recognition software for image analysis oriented towards cones and vessels. The device enables selection of any area of the retina for imaging, and measurements in the same spot can be repeated based on automatically saved coordinates [22–24]. The rtx1 is particularly valuable for evaluating the progression of retinal changes. Further, it is possible to adjust the depth of the retinal region under evaluation, which permits visualization of individual photoreceptors, intraretinal deposits, neurosensory retinal atrophy, lamina cribrosa, microexudations, and microaneurysms [22, 24]. Two computer programs are provided by the manufacturer for the analysis of the examination findings: AOdetect (for the analysis of photoreceptors) and AOdetectArtery (for the analysis of the retinal vasculature).

The available international literature offers few publications on examination findings using the rtx1 in patients with diabetes [18, 25–29]. To our knowledge, there are no publications based on rtx1 measurements regarding changes in the retina in patients with prediabetes. The objective of this study was to analyze cone parameters and retinal microvascular changes in patients with prediabetes and to compare the findings to results from healthy volunteers.

## 2. Methods

Retinal examinations with the rtx1 device were conducted between May and July 2015 at the Department of Ophthalmology, Second Faculty of Medicine, Medical University of Warsaw, located in the Ophthalmic University Hospital in Warsaw. The study protocol was approved by the Bioethical Commission of the Medical University of Warsaw. Each patient received both oral and written information explaining the objective and design of the study, the operating principles of the device, and the examination procedure. In accordance with the Declaration of Helsinki, written informed consent was obtained from all subjects who participated in the study.

### 2.1. Eligibility Criteria

The study group consisted of adult (>18 years, white Europeans) patients with confirmed prediabetes according to the criteria of the American Diabetes Association [8, 9] for IFG and IGT from the Department of Internal Medicine, Diabetology and Endocrinology, Medical University of Warsaw, and age-matched normoglycemic controls (also confirmed after OGTT). All patients provided a medical history to verify the inclusion and exclusion criteria.

**Figure 1:**
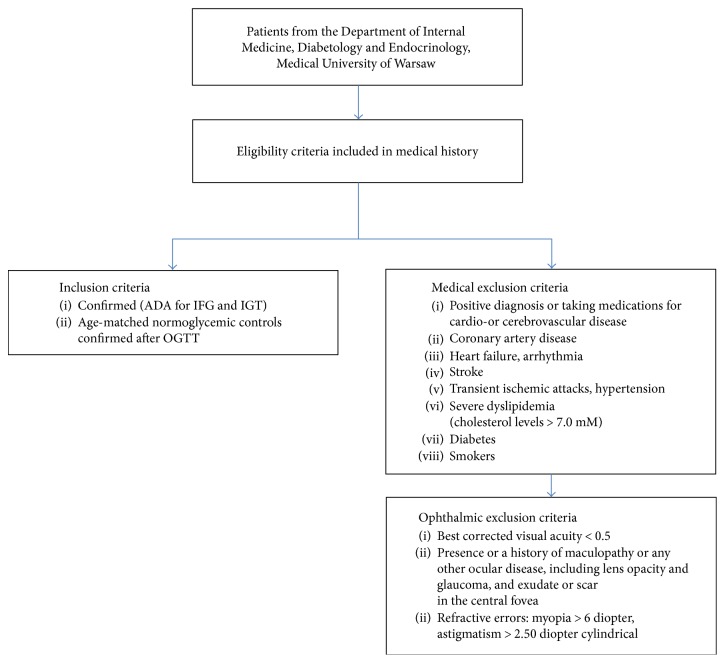
Flow diagram of the study selection process: participant sampling, recruitment, and exclusion. ADA: American Diabetes Association; IFG: impaired fasting glucose; IGT: impaired glucose tolerance; OGTT: oral glucose tolerance test with 75 g of glucose.

Subjects were excluded if they had a positive diagnosis or were taking medications for cardio- or cerebrovascular disease, coronary artery disease, heart failure, arrhythmia, stroke, transient ischemic attacks, atrial hypertension (defined by systolic blood pressure > 140 mmHg and/or diastolic blood pressure > 90 mmHg), severe dyslipidemia (cholesterol levels > 7.0 mM), or diabetes. Subjects who were smokers were also excluded. The ophthalmic exclusion criteria were best-corrected visual acuity less than 0.5; the presence or a history of maculopathy or any other ocular disease, including lens opacity, glaucoma, and exudate or scar in the fovea; and refractive errors for myopia > 6 diopters or astigmatism > 2.50 diopter cylindrical. Eligibility for study participation was confirmed by comprehensive ocular examination. Participant sampling, recruitment, and exclusion are described in the flowchart diagram (Figure 1). Participants were divided into 2 groups: control (healthy subjects) and prediabetic (patients with IFG or IGT and no retinopathy). The control group was built by including only healthy participants with the same exclusion criteria and BMI lower than 25 kg/m^2^.

### 2.2. Prediabetic and Control Groups

An initial group of 43 individuals with IGT or IFG was screened. Thirty-one were excluded because they did not meet the inclusion criteria (20 treated for hypertension, 5 with a history of cardiovascular event, 3 with maculopathy, and 3 with diagnosed primary open-angle glaucoma). The prediabetic group included 12 subjects comprising 9 women (75%) and 3 men (25%). The control group consisted of 22 healthy volunteers 13 women (59%) and 9 men (41%). Age and axial length were not significantly different between the groups. The mean ± standard deviation age in the control group and the prediabetic group was 42.4 ± 14.3 and 52.6 ± 10.0, respectively (*p* = 0.873). The mean axial length of the right eye in the control group was 24.24 ± 1.45 mm, and that of the left eye was 24.12 ± 1.52 mm (Mann–Whitney *U* test, *p* = 0.580). The mean axial length of the right eye in the prediabetic group was 23.78 ± 1.52 mm, and that of the left eye was 23.29 ± 0.63 mm (Mann–Whitney *U* test, *p* = 0.990). Because eyes did not differ in terms of axial lengths, only results obtained from right eyes were included in further analyses. The characteristics of both groups are in shown in Table 1.

### 2.3. rtx1 Examination Procedure

The AO retinal camera (rtx1) was used to acquire images of parafoveal cones and retinal arterioles with a diameter greater than 70 *μ*m in patients from both groups. Each rtx1 examination captured scans of the 4 perifoveal areas of the retina, 3° (approximately 900 *μ*m) off the center of the fovea (temporally, nasally, superiorly, and inferiorly). A 100 × 100 *μ*m sampling window size was chosen. Most of the examinations did not require dilation of the pupils. In isolated cases, in which the width of the pupil was less than 4 mm, one drop of 1% tropicamide was administered.

Images were taken outside the foveolar avascular zone. The best sections of images captured were selected and analyzed using the image processing and recognition software AOdetect (for the analysis of photoreceptors) and AOdetectArtery (for the analysis of the retinal vasculature).

In addition, all subjects had noncontact ocular biometry using the IOL Master (Carl Zeiss Meditec AG, Hennigsdorf, Germany).

### 2.4. rtx1 Parameters Evaluated

The automated algorithm analyses of the photoreceptors included the mean ± standard deviation cone density per square millimeter of the retinal surface and the morphology of the cones in terms of neighborhood (Voronoi domain), with the percentage of cone photoreceptors showing optimal hexagonal (*N* = 6) tiling as well as 5-, 7-, and 8-sided organization and distribution. The dimensional features for the cone mosaic were calculated from the tile reconstruction for identified cone by defying regions based on the Voronoi tile matrix and the junction with the Delaunay triangulation.

In the analyses of retinal vessels, 3 measurements of the retinal arterioles and veins with a size between 70 and 130 *μ*m in temporal superior quadrants were taken. The arithmetic average of these 3 values was taken. Measurements of the vessel diameter (VD), the wall thickness (WT), and the lumen diameter (LD) were recorded, and the wall-to-lumen ratio (WLR) and the cross-sectional area of the vascular wall (WCSA) were then automatically calculated. Vessel diameter (VD) resulted from single arteriolar wall (WT) plus vessel lumen (LD) and single arteriolar wall thickness (WT): VD = WT + (WT + LD). WLR was calculated as WLR = 2·WT/LD. The WCSA of the vascular wall was measured on the basis of vessel diameter and lumen diameter, and the value was obtained automatically from the AO artery detect software.

### 2.5. Medical History of the Prediabetic Group

Recent results of blood parameters were analyzed, including FPG, OGTT with 75 g of glucose, glycated hemoglobin A_1c_ (HbA_1c_), insulin, triglyceride (TG), total cholesterol, low-density lipoprotein cholesterol (LDL), high-density lipoprotein cholesterol (HDL), body mass index (BMI), and the correlation between chosen parameters and cone density and WLR/WCSA ratio.

### 2.6. Statistical Analyses

Descriptive analyses were conducted for all variables that were visually assessed for outliers. Four measurements of cone density in the location of a region of interest (lower, upper, nasal, and temporal quadrants) were collected from each individual. The measurements were collected from both eyes in a subset of the subjects. The Kolmogorov-Smirnov and Shapiro-Wilk tests were used to determine if the parameters were normally distributed. Because the parameters were not normally distributed, we used the Wilcoxon signed-rank test for comparing data from the right and the left sides and the Mann–Whitney *U* test for comparing the prediabetic and control groups.

Simple and multivariate linear regressions were performed to determine the effect of demographic and optical (Age, gender, BMI, and AL) characteristics and blood parameters (FPG, 2-h OGTT, HbA_1c_, Total cholesterol, TG, LDL, and HDL) (independent variables) on parameters WLR and WCSA (dependent variables) measured with the use of rtx1. Potential modifiers were examined in stratified analysis. All probabilities quoted are 2-sided, and a significant *p* value was defined as <0.05.

The Pearson correlation coefficient (*r*) was calculated to examine the linear relationship between 2 continuous variables of WLR and WCSA with the blood parameters and BMI. Statistical analyses were generated with StatSoft Inc. (2014), STATISTICA (data analysis software system), version 12 (http://statistica.io/).

## 3. Results

The best-corrected visual acuity was 1.0 in the control group and ranged from 1.0 to 0.8 (mean 0.94) among patients with prediabetes.

In the prediabetic group, the mean FPG was 102 ± 11 mg/dL (range, 81–118 mg/dL) (5.7 ± 0.6 mmol/L), the mean HbA_1c_ level was 5.56 ± 0.36% (range, 5.0%–6.0%), the mean insulin level was 12.7 ± 12.4 (range, 4.9–37.5), and the mean BMI was 30.4 ± 5.7 (range, 24–41.4). The results of the blood parameters and BMI in the prediabetic and the control groups are presented in Table 1.

### 3.1. Cone Parameters

The mean cone density (DM) was not significantly different between right eyes (RE) and left eyes (LE) (18,935 ± 1713 cells/mm^2^ for RE and 19,694 ± 2339 cells/mm^2^ for LE; *p* = 0.407) (Table 2) and in the 4 retinal quadrants in the prediabetic group (*p* = 0.954) (Table 3). There were also no significant differences in the mean intra eye DM values in the control group (19,900 ± 2375 cells/mm^2^ for RE and 21,435 ± 2494 cells/mm^2^ for LE; *p* = 0.290) (Table 2) and in the 4 retinal quadrants (*p* = 0.188) (Table 3). Results obtained from RE in both groups were chosen for further analysis.

The mean ± SD cone density in the RE in the control and the prediabetic groups was 19,900 ± 2375 cells/mm^2^ and 18,935 ± 1713 cells/mm^2^, respectively (*p* = 0.0928) (Table 3). The cone density was not significantly lower in the prediabetic group compared to the control group at the 900 *μ*m retinal eccentricities. No statistically significant differences were observed between groups in any of cone parameters in four analyzed retinal quadrants.

The mean ± SD interphotoreceptor distance (SM) in the prediabetic and the control groups was 8.02 ± 0.39 and 7.76 ± 0.53 *μ*m, respectively (*p* = 0.106).

The mean percentage ± SD of cones with hexagonal Voronoi tiles (N%6) in the control and the prediabetic groups was 51.2% ± 4.2% and 47.6% ± 4.1%, respectively (*p* = 0.0198) (Table 3). Pentagonal (N%5) and septagonal (N%7) cone shapes were observed in the control and the prediabetic groups by the mean percentage ± SD as 25.1% ± 2.0% and 23.8% ± 1.4% (*p* = 0.0472) and 21.1% ± 0.8% and 20.0% ± 1.6%, respectively (*p* = 0.0138).

**Figure 2:**
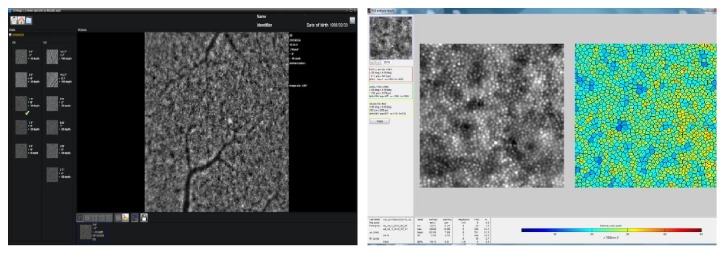
Image of the retina captured with the rtx1 adaptive optics flood-illumination retinal camera with 100 × 100 *μ*m sampling window and visualization of cones using AOdetect by the Voronoi triangulation with a hot color scale. The control group subject's characteristics: male; 27 years old; AL 24.00 mm; DM 23.105 ± 3726 1/mm^2^; SM 7.24 ± 0.72; N%6 51.3%; and number of spots 1444.

The representative image of cone analysis is shown in Figure 2.

### 3.2. Retinal Artery Parameters

The mean WLR and the mean WCSA were not significantly different between RE and LE in the prediabetic and in the control groups. Results obtained from RE in both groups were chosen for further analysis. The mean lumen diameter (LD) and the mean WLR values differed significantly between the prediabetic group and the control group (for LD 94.3 ± 10.9 versus 105.6 ± 14.6 *p* = 0.022 and for WLR 0.29 ± 0.05 versus 0.22 ± 0.04; *p* < 0.001). No statistically significant differences were observed between the prediabetic and the control groups in the mean WCSA (4519 ± 905 versus 4284 ± 982); *p* = 0.490) (Table 4).

### 3.3. Retinal Venule Parameters

No statistically significant differences were found in the analyzed retinal venule parameters between the prediabetic and the control groups (LD, 136.9 ± 17.6 versus 146.3 ± 16.6; WCSA, 2956 ± 487 versus 3500 ± 894; and WLR, 0.097 ± 0.016 versus 0.099 ± 0.020) (Table 4).

**Figure 3:**
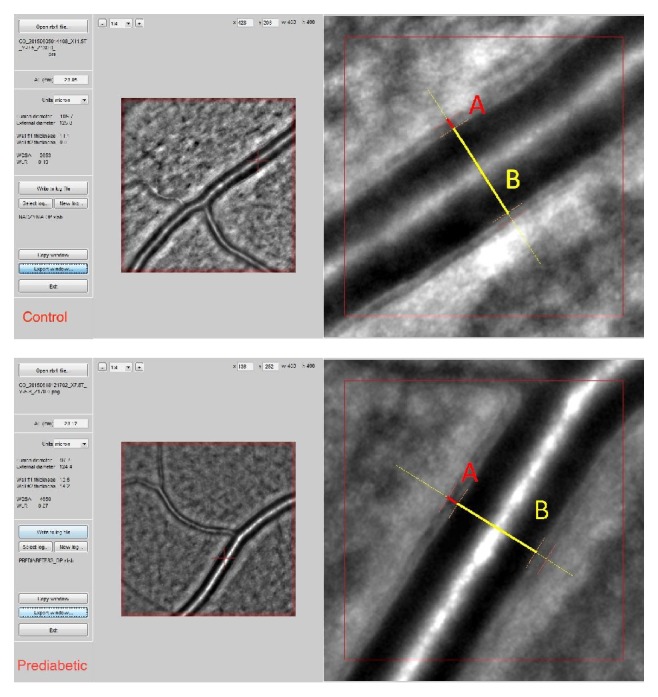
Image of the retinal artery of a patient from the control (female, 58 years old, WLR 0.19) and the prediabetic (female, 51 years old, WLR 0.27) groups (from top to bottom: control and prediabetic) captured with the rtx1 adaptive optics flood-illumination retinal camera with 400 × 400 *μ*m sampling window obtained automatically and wall and lumen visualization using AOdetectArtery, where A is a single arteriolar vessel wall thickness (VW) and B is a vessel lumen diameter (LD).

Representative images from the retinal artery analysis in a patient from the control and prediabetic groups are shown in Figure 3.

### 3.4. Correlation between Cone Parameters and Blood Parameters and BMI in the Prediabetic Group

Correlations were found between cone parameters (DM, SM, percentage of cones with hexagonal Voronoi tiles, Pearson coefficient *r* = −0.986; *p* < 0.05) and the mean level of HbA_1c_ (Pearson coefficient *r* = 0.822 and *r* = −0.770; *p* < 0.05). There were no correlations between cone parameters and the mean BMI and FPG, TG, cholesterol, LDL, and HDL results.

### 3.5. Correlation between Retinal Artery Parameters and Blood Parameters and BMI in the Prediabetic Group

**Figure 4:**
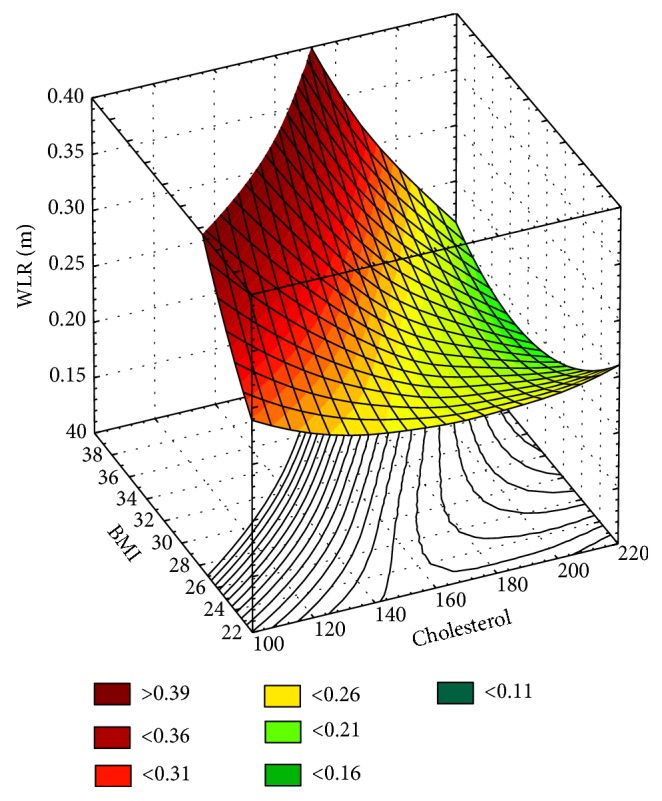
3D surface of linear regression model for WLR dependence on cholesterol and BMI (independent variables) for the prediabetes group.

Correlations were found between WLR and WCSA and the level of total cholesterol (Pearson coefficient *r* = −0.894; *p* < 0.05). There was no correlation between the mean levels of FPG, OGTT, HbA_1c_, TG, LDL, and HDL. In addition, a multivariate regression analysis with dependent variable WLR showed significant and positive correlations between WLR and BMI (*b* = 0.0047 ± 0.0016, *p* = 0.0418) and total cholesterol analyzed together (*b* = −0.0013 ± 0.0002, *p* = 0.0038). In an analysis of both normalized parameters, total cholesterol had a 2-fold higher influence on the WLR than BMI (*b*^∗^ = −1.0057 ± 0.1670 and *b*^∗^ = −0.4935 ± 0.1670; adj. *R*^2^ = 0.854; standard error of estimation 0.021) (Figure 4).

## 4. Discussion

In this case-control study carried out in a sample of people with diagnosed IGT, we analyzed retinal photoreceptor morphology and retinal microvasculature with the new device rtx1. The rtx1 is a microscope with adaptive optics technology, which provides detailed visualization of retinal microstructures. We hypothesized that small changes could be found that were not possible to observe with other currently available methods. Our results suggest that individuals with IGT demonstrate signs of an early dysfunction of retinal arterioles as measured by the WLR. The study group with prediabetes excluded patients with hypertension or other vascular disorders to avoid the influence of these conditions on the retinal vessel parameters.

Increased WLR was found in the prediabetic group, and WLR was also found to be correlated with BMI and total cholesterol in this group. Arterial remodeling is best characterized by increased WLR, and an overall increase in WLR can result from wall thickening, narrowing of the lumen, or a combination of both. Our results are similar to those obtained by other authors, who also described narrowing of arterioles lumen in prediabetes and in early diabetes without signs of retinopathy [12, 30, 31]. The retinal microvasculature provides a direct means of visualizing the systemic microcirculation, and retinal vascular changes reflect the deleterious effects of hyperglycemia on the systemic microcirculation.

The majority of studies suggest that patients with a short duration of diabetes (<5 years) show constriction of arteries and small retinal arterioles as well as decreased retinal blood flow [19, 21, 32, 33]. The results of the AusDiab study also confirmed that wider arteriolar wall caliber is a specific and effective indicator of diabetic microvascular dysfunction independent of blood pressure, glycemic control, and other retinopathy risk factors and serves as a “prepathology” marker for DR [31]. These results support our findings of increased WLR and no changes in venule parameters in patients with IGT compared to age-matched healthy controls. These findings are also in agreement with the hypothesis that the autoregulation dysfunction occurs first in arterioles then in venules [30]. Conversely, retinal venular dilatations are thought to represent a later sign of DR and its progression [34]. Laser Doppler techniques have also revealed reduced blood flow in retinal vessels in diabetic patients even without retinopathy [35]. At the early stages of prediabetes or diabetes, hypoxia leads to hypoperfusion and low-grade chronic inflammation of the retinal microvasculature, which initially results in capillary dysfunction [4, 5, 7]. A strong correlation between chronic inflammation and signs of isolated retinopathy in people without diabetes was also confirmed in the Hoorn Study [36]. This correlation may explain the pathophysiology of microvascular changes in prediabetes [36]. In contrast to our results are conclusions from the Rotterdam Study and the Blue Mountain Eye Study in which decreased arteriovenous ratio was found to depend on venular dilatation not arteriolar narrowing in patients with IGT and early diabetes [37, 38].

Limitations in currently available imaging instruments that caused the main purpose of investigators were conducted on the microvascular changes in prediabetes and diabetes. The in vivo evaluation of the structures of neuronal retinal cells was not previously possible, but adaptive optics retinal cones mosaic imaging now offers a noninvasive method [28]. Aside from the assessment of retinal vessels, we used the rtx1 microscope to analyze and compare cone density and regularity in age-matched healthy volunteers and patients with prediabetes. Our findings indicate that cone density is not affected by the impaired glucose tolerance in comparison with the results from healthy retinas examined in the same localization. The interphotoreceptor distance also did not show a statistically significant difference between the 2 groups. However, some studies have revealed a decline in the photoreceptor counts in patients with type 1 diabetes even at early stages of DR versus healthy controls. The decrease in cone density was associated with the duration of diabetes or serum HbA_1c_ levels [27]. We found no significant differences in any of analyzed parameters between groups. Lombardo et al. [28] showed 6% lower cone density and differences in the percentage of hexagonal cones in diabetic eyes without DR than in the control groups. However, they indicated that this result should not be considered clinically significant because of the variability of cone density across a normal adult population [28]. Recently, Tan et al. investigated the retinal periphery (7° from the fovea) of 29 adolescents and young adults with type 1 diabetes at an early stage and no signs of DR on fundoscopy; no differences in cone density were found in comparison to age-matched controls. However, this study was limited to relatively younger patients with no DR [39]. These results are similar to our findings. We analyzed cone density further than Lombardo et al. [28], to approximately 3° from the fovea. The results of our work and of other authors confirm that cone density alone cannot clearly identify the early pathological changes of the parafoveal cone mosaic in patients with prediabetes [28, 39].

### 4.1. Study Limitations

Our study was limited in that the case and control sample sizes were relatively small because of the restricted exclusion criteria. Consequently, the results should be treated with caution because there might be smaller but clinically relevant differences that in the study did not have the statistical power to identify.

## 5. Conclusions

In conclusion, the adaptive optics retinal imaging accurately identified the cone mosaic and retinal microvasculature in patients with prediabetes. Abnormalities found in rtx1 examinations should be considered as early microvascular signs of arteriolar dysfunction, before the progression from IGT to diabetes. These changes may be a strong risk factor for cardiovascular changes, which are also more common in this group of patients. The retinal image analysis with rtx1 offers a novel noninvasive measurement of early changes in the vasculature that are not detectable on routine clinical examination. This measurement may allow the identification of individuals at risk of diabetes, hypertension, and the associated complications. Further analysis on a large cohort of patients would be helpful to understand the potential of adaptive optics-based imaging biomarkers in patients with prediabetes and diabetes as well as other vascular disorders.

## Figures and Tables

**Table 1:** Group characteristics and results of the blood parameters and BMI in the groups.

	Total	Prediabetic	Control	
	*n* (%)	*n* (%)	*n* (%)	
*Number of subjects*	34 (100)	12 (35)	22 (65)	
Women	22 (65)	9 (75)	13 (59)	
*Characteristics*	m ± SD	m ± SD	m ± SD	*p* value^†^
Age (years)	52.1 ± 11.9	52.6 ± 10.0	42.4 ± 14.3	0.8733
BMI (kg/m^2^)	26.1 ± 3.9	30.4 ± 5.7	25 ± 2	0.0030
AL (mm)	24.17 ± 1.52	23.78 ± 1.52	24.24 ± 1.45	0.3568
*Blood parameters*	m ± SD	m ± SD	m ± SD	*p* value^†^
FPG (mg/dL)	91 ± 10	102 ± 11	88 ± 6	0.0144
FPG (mmol/L)	5.0 ± 0.7	5.7 ± 06	4.8 ± 0.6	0.0038
2-h OGTT (mg/dL)	107 ± 27	141 ± 23	96 ± 17	0.0001
HbA_1c_ (%)	5.46 ± 0.26	5.56 ± 0.36	5.43 ± 0.22	0.4227
Insulin (mIU/mL)	8.4 ± 6.5	12.7 ± 12.4	7.1 ± 3.1	0.3215
Total cholesterol (mg/dL)	117 ± 24	174 ± 41	168 ± 17	0.6142
TG (mg/dL)	99 ± 33	115 ± 44	94 ± 28	0.2591
LDL (mg/dL)	114 ± 27	117 ± 27	113 ± 27	0.7994
HDL (mg/dL)	51 ± 10	52 ± 10	51 ± 10	0.6780

^†^Mann–Whitney *U* test. m: mean; SD: standard deviation; AL: mean axial length; FPG: fasting plasma glucose; 2-h OGTT: oral glucose tolerance test with 75 g of glucose; HbA_1c_: glycated hemoglobin A_1c_; TG: triglyceride; LDL: low-density lipoprotein cholesterol; HDL: high-density lipoprotein cholesterol; BMI: body mass index.

**Table 2:** The mean cone density in RE and LE in both groups (*n* = 12).

Group	m ± SD DM RE	m ± SD DM LE	*p* value^†^
Prediabetic	18,935 ± 1713	19,694 ± 2339	0.4068
Control	19,900 ± 2375	21,435 ± 2494	0.2895
Total	19,286 ± 1981	20,419 ± 2460	0.1530

^†^ *t*-test. m: mean; SD: standard deviation; DM: cone density; RE: right eye; LE: left eye.

**Table 3:** The mean cone density in 4 retinal quadrants and characteristic of cone parameters in the groups.

Parameters	Total	Prediabetes	Control	*p* value^†^
Quadrants	m ± SD	m ± SD DM	m ± SD DM	
DM (T)	19,778 ± 2901	19,205 ± 2803	20,851 ± 3013	0.0899
DM (N)	20,311 ± 3227	19,930 ± 3737	20,838 ± 2393	0.4487
DM (S)	19,009 ± 2742	19,030 ± 2198	19,304 ± 3610	0.6283
DM (I)	19,850 ± 2851	19,373 ± 2398	20,970 ± 3299	0.0708
4 quadrants' *p* value^‡^	0.6313	0.9536	0.19833	
DM (1/mm^2^)	19,686 ± 2195	19,188 ± 1919	20,491 ± 2443	0.0928^†^
SM (1/mm^2^)	7.92 ± 0.46	8.02 ± 0.39	7.76 ± 0.53	0.1062^†^
N%5	24.6 ± 1.9	25.1 ± 2.0	23.8 ± 1.4	0.0472^§^
N%6	49.0 ± 4.5	47.6 ± 4.1	51.2 ± 4.2	0.0198^†^
N%7	20.7 ± 1.3	21.1 ± 0.8	20.0 ± 1.6	0.0138^§^
N%8	3.1 ± 1.4	3.4 ± 1.5	2.7 ± 1.2	0.1898^§^

^†^ *t*-test. ^‡^Friedman ANOVA test. ^§^Mann–Whitney *U* test. m: mean; SD: standard deviation; DM: cone density. Quadrants—T: temporal; N: nasal; S: superior; I: inferior; N%*n*: percentage distribution of *n*-polygon cone packing as a function of the neighborhood; N%5: pentagonal shape; N%6: hexagonal shape; N%7: septagonal shape; N%8: octagonal shape.

**Table 4:** Characteristic of retinal artery and venule parameters in the prediabetic and control groups (*n* = 12 and 22).

Parameters	m ± SD Prediabetic	m ± SD Control	*p* value
LD artery (*μ*m)	94.3 ± 10.9	105.6 ± 14.6	0.022^†^
WLR artery	0.29 ± 0.05	0.22 ± 0.04	<0.001^†^
WCSA artery	4519 ± 905	4284 ± 982	0.490^†^
LD venule (*μ*m)	136.9 ± 17.6	146.3 ± 16.6	0.136^†^
WLR venule	0.097 ± 0.016	0.099 ± 0.020	0.848^‡^
WCSA venule	2956 ± 487	3500 ± 894	0.055^†^

^†^ *t*-test. ^‡^Mann–Whitney *U* test. m: mean; SD: standard deviation; DM: cone density; LD: lumen diameter; WLR: wall-to-lumen ratio; WCSA: wall cross-sectional area.
